# Constraint-Induced Movement Therapy in the Rehabilitation of Adults After Stroke: An Umbrella Review

**DOI:** 10.3390/jcm15062451

**Published:** 2026-03-23

**Authors:** José Conchillo-Liria, Iván Cavero-Redondo, Alicia Saz-Lara, Nerea Moreno-Herraiz, Candela Calvo-Utrilla, Ana González-Collado, Iris Otero-Luis

**Affiliations:** 1 CarVasCare Research Group, Instituto de Biomedicina/Facultad de Enfermería de Cuenca, Universidad de Castilla-La Mancha, 16071 Cuenca, Spain; pepe_pcl@hotmail.com (J.C.-L.); alicia.delsaz@uclm.es (A.S.-L.); nerea.moreno@uclm.es (N.M.-H.); candela.calvo@uclm.es (C.C.-U.); ana.gonzalezcollado@uclm.es (A.G.-C.); iris.otero@uclm.es (I.O.-L.); 2 Faculty of Nursing, Universidad de Castilla-La Mancha, 02006 Albacete, Spain

**Keywords:** stroke, upper limb, constraint-induced movement therapy, occupational therapy

## Abstract

**Background/Objectives**: Stroke is among the leading causes of disability in adults, as hemiparesis affects motor function and daily activities. Constraint-induced movement therapy (CIMT) has proven effective in functional recovery through intensive use of the affected limb. This study aimed to assess the impact of CIMT on upper limb (UL) rehabilitation in stroke patients, with a focus on motor recovery, integration into activities of daily living (ADLs), and overcoming clinical implementation barriers. **Methods**: A systematic review was conducted by searching PubMed, Scopus, and Web of Science from their inception to March 2026. Systematic reviews and meta-analyses evaluating the effectiveness of CIMT in adult patients after stroke were included. The outcome variables included motor function, movement quality, independence in ADLs, and quality of life (QoL). **Results**: Twenty-five systematic reviews and sixteen meta-analyses were included. The participants were adults who had suffered a stroke at acute, subacute, or chronic stages and were aged between 18 and 95 years. With respect to upper limb motor function, ten studies reported statistically significant results in favor of CIMT. With respect to ADLs, four studies reported significant differences in favor of CIMT, with strong effects in intensive interventions. With respect to QoL, three studies reported significant improvements after the intervention. **Conclusions**: The results of this umbrella review support the effectiveness of CIMT in UL rehabilitation after stroke, especially in the subacute and chronic phases. CIMT, alone or in combination with adjuvant therapies, contributes to improving motor function, independence in ADLs, and QoL in patients.

## 1. Introduction

Stroke is among the leading causes of acquired disability in the adult population worldwide. This neurological disorder, caused by an alteration in cerebral blood flow, affects approximately 12 million people per year, causes 6.5 million deaths and leaves millions of survivors with significant sequelae [1]. Stroke can be classified into two main types: ischemic, which accounts for 85% of cases, and hemorrhagic, which accounts for the remaining 15% of cases [2].

According to the World Stroke Organization, more than 101 million people worldwide are currently living with the effects of stroke. Recent estimates indicate that approximately 68 million adults aged 20 years or older have experienced ischemic stroke, and the lifetime risk of stroke among individuals over 25 years of age is approximately one in four globally [3,4]. In Spain, the prevalence of stroke in adults ranges from 500 to 800 cases per 100,000 inhabitants [3]. It is the leading cause of physical disability and one of the main causes of functional dependency [5]. Among the most common sequelae, hemiparesis affects mobility and functionality on one side of the body, significantly impairing the ability to perform ADLs [6]. This condition is often associated with the learned nonuse phenomenon, which occurs when patients stop using the affected limb because of previous failed attempts [3].

Rehabilitation after stroke aims to reduce disability and improve functional independence through various therapeutic strategies. In this context, CIMT has emerged as an evidence-based intervention designed to compensate for learned nonuse and promote functional recovery. CIMT involves restricting the use of the unaffected limb while encouraging intensive use of the affected limb through structured, repetitive activities, with the aim of stimulating neuroplasticity and restoring functional motor pathways [7].

To assess the effectiveness of CIMT in patient recovery, several standardized tools are used to measure improvements in motor function, independence, and QoL. Among these tests, the Fugl–Meyer assessment (FMA) is used to evaluate upper limb motor recovery, whereas the Wolf Motor Function Test (WMFT) is used to assess movement speed and quality [7]. The action research arm test (ARAT) measures manual dexterity and upper limb functionality, and the Barthel index helps determine the level of independence in ADLs [3]. Additionally, self-reported QoL questionnaires provide a comprehensive view of the impact of therapy on overall well-being [1].

Despite its proven efficacy, the implementation of CIMT in clinical practice presents several challenges, such as the time and effort required for both the patient and the therapist, the need to meet specific inclusion criteria to maximize effectiveness, and the lack of specialized resources in certain rehabilitation settings. These challenges highlight the importance of adapting and optimizing existing protocols to increase accessibility and feasibility [1,7].

Numerous studies have shown that CIMT can lead to significant improvements in both the quality and quantity of movement of the affected limb, as well as in the functional independence of patients [6,8,9]. A meta-analysis revealed that CIMT is highly effective in the rehabilitation of patients with hemiparesis after stroke [10]. Similarly, another study highlighted its ability to produce sustained medium- and long-term effects, facilitating better reintegration into ADLs [11]. Moreover, systematic reviews have provided evidence of the functional benefits of CIMT in both the subacute and chronic phases of stroke [1,3,7]. More recent studies have compared CIMT with other interventions and concluded that, despite its high intensity, CIMT offers superior results in terms of motor recovery and participation in ADLs [2,4].

The scientific literature includes several systematic reviews exploring the effectiveness of CIMT at different stages of stroke recovery, as well as its comparison with other rehabilitation interventions. These reviews have focused mainly on the clinical effectiveness of the intervention from a medical or physiotherapeutic perspective, emphasizing quantitative parameters of functional recovery. However, this umbrella review offers a novel approach that provides a more holistic understanding of the therapeutic impact, including variables such as patient-perceived QoL, treatment adherence, and applicability in both clinical and home settings. For these reasons, this umbrella review aimed to analyze the evidence for CIMT in the rehabilitation of upper limbs in adult patients after stroke, highlighting its impact on motor function, quality of movement, independence in ADLs, and QoL.

## 2. Materials and Methods

This umbrella review was conducted in accordance with the Preferred Reporting Items for Systematic Reviews and Meta-Analysis (PRISMA) guidelines [12] and the Cochrane Handbook for Systematic Reviews of Interventions [13]. This study was registered in PROSPERO (registration number: CRD420251235617) (Table S1).

### 2.1. Search Strategy

A comprehensive search strategy was used to identify relevant literature in the PubMed, Scopus, and Web of Science databases from their inception to March 2026. For this purpose, the following terms were combined: stroke, chronic stroke, subacute stroke, hemiparesis, upper limb, upper extremity, constraint-induced movement therapy, repetitive task practice, CIMT, QoL, motor activity, participation, ADLs, and motor function. These terms were integrated via Boolean operators AND/OR and structured according to the PIO model (population, intervention, outcome) to identify studies assessing the effectiveness of CIMT in upper limb rehabilitation in adult patients after stroke. The following search strategy was used in Pubmed: (“Stroke”[Mesh] OR “Stroke”[Title/Abstract] OR “Hemiparesis”[Title/Abstract] OR “Paresis”[Mesh]) AND (“Upper Extremity”[Mesh] OR “Upper Limb”[Title/Abstract]) AND (“Constraint-Induced Therapy”[Mesh] OR “Constraint-Induced Movement Therapy”[Title/Abstract] OR “CIMT”[Title/Abstract] OR “Repetitive Task Practice”[Title/Abstract]) AND (“Quality of Life”[Mesh] OR “Activities of Daily Living”[Mesh] OR “ADL”[Title/Abstract] OR “Patient Participation”[Mesh] OR “Motor Skills”[Mesh]) AND (systematicreview[Filter] OR metaanalysis[Filter]) Additionally, the reference lists of the included systematic reviews and meta-analyses were manually screened to identify potentially eligible studies (Tables S2 and S3).

### 2.2. Eligibility Criteria

The inclusion criteria were as follows: (i) adults (>18 years) with a clinical diagnosis of ischemic or hemorrhagic stroke; (ii) intervention: CIMT in the context of rehabilitation after stroke; (iii) outcomes: use of assessment tools such as the Motor Activity Log (MAL), Functional Independence Measure (FIM), and Stroke-Specific Quality of Life Scale (SS-QOL); and (iv) study design: systematic reviews and meta-analyses. In contrast, the following exclusion criteria were used: (i) studies not specifically focused on upper limb rehabilitation following stroke; (ii) studies that included robotic devices as part of the rehabilitation process; (iii) studies in which the primary outcome did not include upper limb functionality, QoL, or performance in ADLs; and (iv) publications not included in peer-reviewed scientific journals.

Outcome eligibility was defined by clinical constructs rather than specific scales. In accordance with the ICF framework, studies were included if they utilized any validated instrument to assess upper-limb motor function, ADL performance, or quality of life [14,15]. Studies involving robotic devices were excluded to ensure ecological validity and the transferability of results to the patient’s natural environment. Unlike clinical robotic systems, pure CIMT focuses on unassisted motor effort during ADLs. Excluding robotic support allows for a more accurate assessment of how intensive practice translates to functional independence in home-based contexts, such as dressing or bathing, where such technology is unavailable [14].

**Figure 1:**
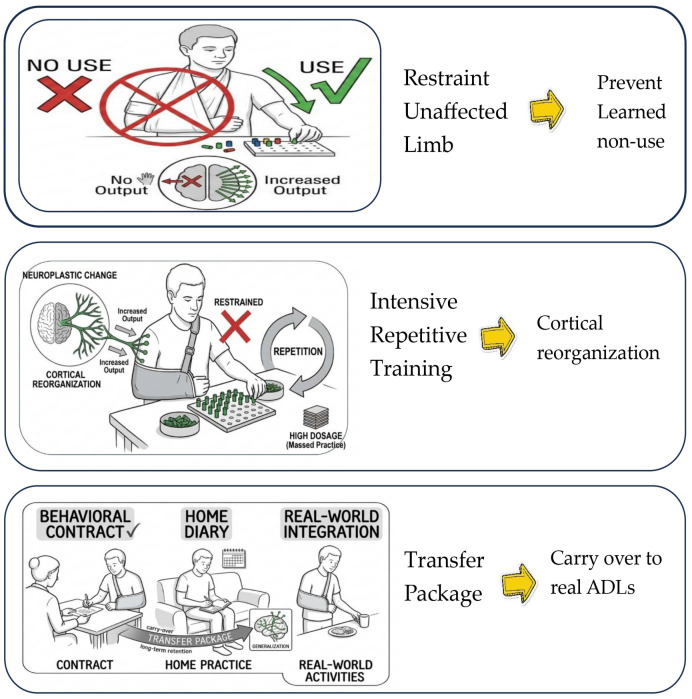
Three Core Pillars of the CIMT [16].

To ensure consistency in the evidence synthesis, the CIMT was defined on the basis of the three core pillars of the protocol: (1) Restraint of the unaffected upper limb (using a mitt, sling, or splint), (2) intensive repetitive task practice, and (3) behavioral shaping [16]. The included reviews included both “Signature CIMT” (typically involving 6 h of daily training and restraint for 90% of waking hours) and ‘Modified CIMT’ (mCIMT), which utilizes lower intensities (e.g., 2–3 h per day) over a longer duration. Reviews focusing solely on bimanual training without a clear forced-use component of the paretic limb were excluded to maintain a theoretical focus on the ‘Transfer Package’ and ‘cortical reorganization’ [17] (Figure 1).

### 2.3. Data Extraction

Two ad hoc tables were created, one for systematic reviews and another for meta-analysis, to extract and analyze the following information from the selected studies: (1) reference (first author and year of publication); (2) number of included studies; (3) type and phase of stroke; (4) number of subjects; (5) age range; (6) intervention; (7) comparison; (8) duration of the intervention (in weeks); (9) study variables; (10) assessment tools; and (11) methodological quality assessment via the AMSTAR 2 scale.

### 2.4. Methodological Quality Assessment

The methodological quality of the included studies was assessed via the AMSTAR 2 tool [18], which evaluates the risk of bias in systematic reviews. This tool consists of 16 domains that address key methodological aspects, each of which is answered “yes”, “no”, “cannot answer”, or “partial yes”. According to the responses provided, studies were classified into different quality levels: high quality when there was no weakness or only one noncritical weakness; moderate quality when there was more than one noncritical weakness; low quality when there was one critical weakness with or without additional noncritical weaknesses; and critically low quality when there was more than one critical weakness, with or without additional weaknesses.

### 2.5. Assessment of the Quality of Evidence

The Grading of Recommendations, Assessment, Development, and Evaluation (GRADE) tool was used to assess the evidence and support the recommendations [19]. The results were rated as high, moderate, low, or very low quality on the basis of study design, risk of bias, inconsistency, indirectness, imprecision, and publication bias.

The selection of studies, data extraction, and assessment of methodological quality were performed independently by two reviewers (J.C.-L. and I.O.-L.). Discrepancies were resolved by consensus or, if necessary, by consulting a third reviewer (I.C.-R.).

### 2.6. Overlap of Primary Studies

To evaluate the degree of redundancy across the included systematic reviews, the corrected covered area (CCA) was utilized to construct a citation matrix to cross-reference primary studies with their corresponding reviews. The CCA was derived using the following equation: (N − r)/[(r × c) − r], where denotes the aggregate number of publications, indicates the count of unique primary studies, and represents the total number of reviews; subsequently, the resulting overlap was categorized as slight (0–5%), moderate (6–10%), high (11–15%), or very high (>15%).

### 2.7. Data Analysis

The estimates of the primary outcomes reported in each study are described and graphically represented in a forest plot, which presents the standardized mean differences (SMDs) with their respective 95% confidence intervals (CIs) and heterogeneity (I2) to evaluate the effectiveness of CIMT in upper limb rehabilitation in adult patients after stroke. The forest plot included motor function measured with the MAL tool, performance in ADLs assessed with the FIM tool, and QoL, which was determined via the SS-QOL scale. The figure was generated via Stata SE, version 15 (StataCorp, College Station, TX, USA).

## 3. Results

### 3.1. Baseline Characteristics

**Figure 2:**
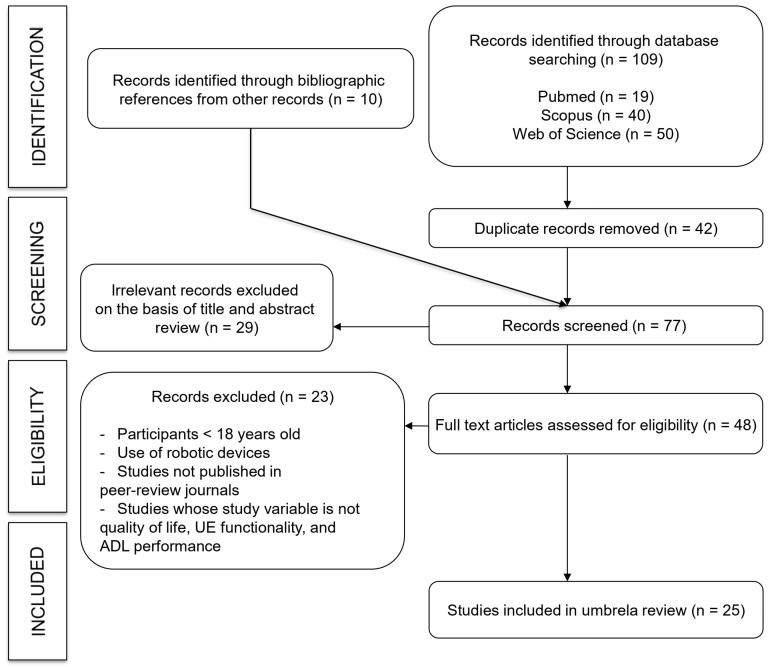
Flowchart: Search strategy.

A total of 119 records were identified (109 identified through database searching, and 10 identified through bibliographic references from other records). After 42 duplicates were removed, 77 studies remained. Following title and abstract screening, 29 studies were excluded. A total of 48 full-text studies were assessed for eligibility, of which 23 were excluded because they did not meet the inclusion criteria. Finally, 25 systematic reviews [1,2,3,4,5,6,7,8,9,10,11,20,21,22,23,24,25,26,27,28,29,30,31,32,33] and 16 meta-analyses [3,4,6,7,9,10,11,25,26,27,28,29,30,31,32,33] were included in the umbrella review (Figure 2).

Two tables were created to show the characteristics of the included studies: one for systematic reviews (Table 1) and one for meta-analysis (Table 2). The reviewed studies included between 2 and 347 randomized controlled trials (RCTs) and were published between 2005 and 2025. The number of participants ranged from 106 to 25,275, all of whom were adults who had experienced ischemic or hemorrhagic stroke. The study participants were aged between 18 and 95 years and were at different phases of stroke recovery: acute [1,2,5,7,8,9,11,22,23,26,27,29,30,32,33], subacute [1,2,5,6,7,8,9,11,22,23,24,26,27,28,29,30,31,32,33], or chronic [1,2,3,4,5,6,7,8,11,21,22,23,24,26,27,28,30,31,32,33].

Intervention durations ranged from 2 to 52 weeks [2,3,4,6,8,9,10,11,20,22,23,24,25,26,27,28,29,30,32,33]. In most studies, the experimental groups (EGs) received CIMT as the main rehabilitation intervention [4,9,20,22,25,27,29,30,32,33]. CIMT is occasionally combined with other therapies [1,5,6,7,8,21,24,26,28,31]. In comparison, the control groups (CGs) received conventional therapy or no intervention [1,2,4,5,7,8,9,10,11,20,21,22,23,24,25,26,27,28,29,30,32,33].

To assess motor functionality and movement quality of the upper limb, the most frequently used tool is the MAL [1,3,4,5,6,8,9,20,21,22,25,26,27,28,29,30,32,33]. Other commonly used assessment tools are the ARAT, FMA, and WMFT [1,2,3,4,5,6,8,9,10,11,20,22,23,25,26,27,28,29,30,32,33].

The scales most commonly used to assess independence in ADLs are the Barthel Index (BI) and the FIM [1,2,5,10,20,21,22,25,26,30,32,33]. Only a few studies have evaluated QoL, typically using tools such as the Short-Form 36 (SF-36), the Short-Form 12 (SF-12), the Stroke Impact Scale (SIS), or the SS-QOL [2,3,5,7,8,21,24,28,31].

### 3.2. Methodological Quality Assessment and GRADE

The methodological quality of the included studies was assessed via the AMSTAR 2 tool. Among the systematic reviews analyzed, 11, 11% were classified as low quality, 66, 67% as moderate quality, and 22, 22% as high quality (Table S4). Among the meta-analyses analyzed, 0% were classified as low quality, 62.5% as moderate quality, and 37.5% as high quality (Table S5).

The certainty of the evidence according to the GRADE tool for upper limb motor function was low or very low in 23 studies because of factors such as high risk of bias, substantial heterogeneity, wide confidence intervals, and suspected publication bias, limiting the confidence in the results despite their potential clinical relevance (Table S6).

The GRADE table for performance in ADLs showed very low certainty for all included studies, owing to a high risk of bias, considerable heterogeneity, imprecision (wide CIs), and publication bias. Despite some important and clinically relevant effects, methodological limitations make it difficult to apply these results (Table S7).

The GRADE table for QoL showed very low certainty in all studies because of bias, moderate to high heterogeneity, a limited number of studies, and wide confidence intervals (Table S8).

### 3.3. Overlap of Primary Studies

To enhance the clinical and statistical rigor of this synthesis, a comprehensive overlap analysis was performed. Recognizing that the included systematic reviews frequently employed disparate assessment instruments for identical clinical constructs, the CCA was calculated independently for the three primary scales most prevalent in the literature: the MAL for motor function, the FIM for ADL performance, and the SS-QOL for quality of life. This granular methodology was adopted to prevent the potential dilution of redundancy data. Furthermore, it ensures that the certainty of evidence is not overstated for any specific outcome, maintaining a high standard of analytical precision across the different clinical domains.

Regarding motor function (MAL), the overlap analysis encompassed 19 systematic reviews containing 142 unique primary studies (r). The cumulative frequency of these studies across the selected reviews reached 395 occurrences (N). Based on these parameters, the calculated CCA was 9.9%, which signifies a moderate degree of overlap according to established interpretation thresholds.

Regarding the assessment of ADL performance via the FIM, the overlap analysis incorporated 10 systematic reviews consisting of 68 unique primary studies (r). The cumulative frequency of these studies across the included reviews was 112 occurrences (N). Consequently, the CCA was determined to be 7.1%, indicating a moderate degree of overlap within this specific clinical construct.

Finally, the assessment of quality of life using the SS-QOL involved an overlap analysis of 3 systematic reviews, which encompassed 24 unique primary studies (R). The cumulative frequency of these studies across the reviews was 31 occurrences (N). This yielded a CCA of 14.5%, a value that, according to established benchmarks, represents a high degree of overlap.

### 3.4. Data Synthesis

In the analysis of upper limb motor function using the MAL tool, 10 of the 14 studies reported statistically significant results. The studies that showed the greatest effect were those by Corbetta et al. (SMD: 0.44; 95% CI: 0.03, 0.84) [10]; De Azevedo et al. (SMD: 0.53; 95% CI: 0.40, 0.66) [26]; Etoom et al. (SMD: 0.56; 95% CI: 0.30, 0.81) [27]; Hestetun-Mandrup et al. (SMD: 0.09; 95% CI: −0.15, 0.34) [28]; McIntyre et al. (SMD: 0.13; 95% CI: 0.02, 0.37) [4]; and Sánchez et al. (SMD: 0.33; 95% CI: −0.04, 0.42) [3]; and Thrane et al. (SMD: 0.51; 95% CI: 0.30, 0.73) [33]. Heterogeneity ranged from low (I2 = 0%) to high (I2 = 96.17%).

In the analysis of ADL performance, the FIM tool revealed statistically significant results: Kaneko et al. (SMD: 0.15; 95% CI: −0.01, 0.32) [6]; Pulman et al. (SMD: 0.03; 95% CI: −0.09, 0.16) [31]; and Thrane et al. (SMD: 0.23; 95% CI: −0.24, 0.71) [33]. Heterogeneity ranged from low (I2 = 0%) to high (I2 = 91.2%).

**Figure 3:**
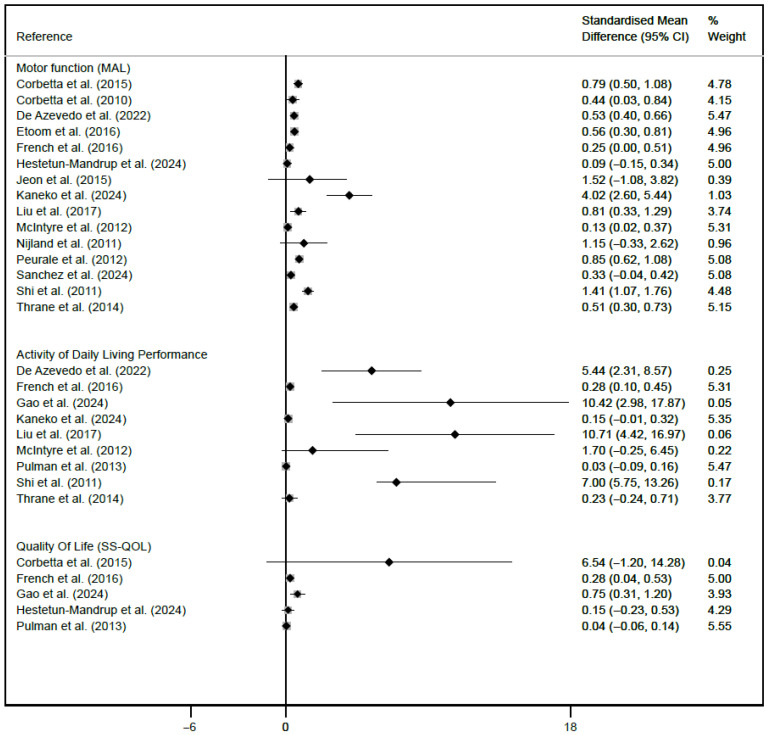
Forest plot for the effect of Constraint-Induced Movement Therapy on motor function, daily living performance and quality of life in people after ictus.

Regarding QoL assessed via the SS-QOL scale, several studies have shown statistically significant results in favor of CIMT: Gao et al. (SMD: 0.75; 95% CI: 0.31, 1.20) [7]; Hestetun-Mandrup et al. (SMD: 0.15; 95% CI: −0.23, 0.53) [28]; and Pulman et al. (SMD: 0.04; 95% CI: −0.06, 0.14) [31]. Heterogeneity ranged from low (I2 = 0%) to moderate (I2 = 42%) (Figure 3).

## 4. Discussion

This umbrella review aimed to analyze the available scientific evidence on the effectiveness of CIMT for upper limb rehabilitation in adult patients after stroke. Overall, the review shows that CIMT, both in its classical and modified forms, contributes significantly to improvements in motor function, performance of ADLs, and perceived QoL, especially when assessed with validated instruments such as the FMA, FIM, and SS-QOL, respectively.

The findings of this review are consistent with previous evidence supporting the concept that CIMT induces neuroplastic changes that lead to improvements in upper limb motor function [3,26,29]. Patients who had experienced significant improvements in strength, coordination, and range of motion in the affected limb, the results of which are reflected in previous reviews [3,7,33]. The combination of CIMT with complementary therapies, such as functional electrical stimulation or mirror therapy, proved particularly effective, which aligns with the findings of previous studies suggesting that multimodal approaches can amplify results [1,5,6,8,24].

With respect to the performance of ADLs, the results confirmed that CIMT is effective at increasing functional independence, as demonstrated by improvements in BI and ARAT scores [2,10,26,29,30,32,33]. Integrating CIMT with additional strategies, such as telerehabilitation, appeared to promote better adherence and functional outcomes, findings that have also been reported in other studies [3,28]. The personalization of the intervention on the basis of the stroke phase and initial severity is a commonly reported recommendation, with general agreement that patients in the subacute and chronic phases are more likely to achieve significant improvements in ADLs [1,4,5,22,26,30,32,33].

In the context of QoL, this review supports previous findings that CIMT can provide psychological and emotional benefits [7]. Patients receiving CIMT-based interventions reported greater autonomy and well-being, a trend that has been consistently observed in previous studies [2,7,27,29]. Furthermore, combining CIMT with complementary therapies, such as functional electrical stimulation or mirror therapy, appears to increase patient motivation and engagement, which are critical factors for long-term adherence [1,6,8,26]. The inclusion of telerehabilitation, especially in settings with limited healthcare access, further expands the potential of these interventions [3,28].

**Figure 4:**
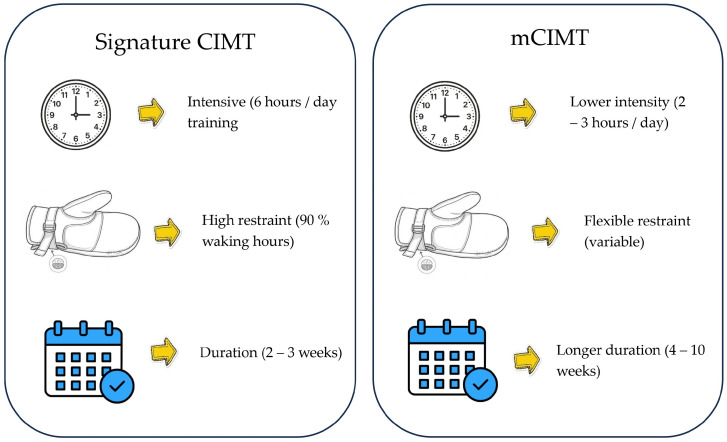
Overview of CIMT Protocols: ‘Signature’ vs. Modified [16].

As illustrated in Figure 4, there is a clear distinction between “Signature CIMT” and “Modified CIMT” (mCIMT) regarding daily dosage, restraint duration, and total program length. While intensive protocols (6 h/day) are traditionally associated with robust cortical reorganization, our synthesis suggests that mCIMT protocols (2–3 h/day) also yield clinically meaningful improvements in upper limb function. This highlights the importance of the “transfer package” and repetitive task practice over the mere duration of physical restraint. Consequently, the choice of protocol should be tailored to the patient’s clinical stage and tolerance, acknowledging that even lower intensity “modified” versions (when consistently applied) effectively counteract “learned nonuse” [16,17].

On the basis of the synthesized evidence, the clinical application of CIMT, combined with complementary therapies, must move away from “one-size-fits-all” models toward personalized stratification according to the stroke phase and severity [6,26]. Our findings suggest that maximizing efficacy requires adapting the intervention intensity to the patient’s stroke phase and motor severity while prioritizing patient-centered goals. To address methodological heterogeneity, clinicians should balance intensive practice with patient tolerance, ensuring that a robust “transfer package” (e.g., behavioral contracts) is implemented to bridge the gap between clinical benefits and real-world independence in ADLs. Furthermore, integrating digital innovations such as telerehabilitation can overcome geographical barriers and ensure continuity of care. Ultimately, a holistic, interdisciplinary approach that considers both physical recovery and psychosocial factors, including motivation and quality of life, is essential for the successful long-term integration of the affected upper limb [28]. Moreover, the potential applicability of CIMT to other neurological conditions, such as cerebral palsy or multiple sclerosis, supports the need for an interdisciplinary approach that considers the physical, emotional, and social dimensions of rehabilitation [3].

Although this umbrella review provides significant evidence for the effectiveness of CIMT, certain limitations should be considered. First, the substantial methodological heterogeneity among the included studies, in terms of study designs, assessment tools, and intervention protocols, complicates direct comparison. Second, many studies had small sample sizes, limiting their statistical power and generalizability. Third, most trials did not assess the long-term sustainability of benefits owing to short follow-up periods, preventing conclusions about the persistence of therapeutic effects. Fourth, wide variation in intervention characteristics, including intensity, duration, and therapeutic combinations, could influence outcome differences. Fifth, while the search was restricted to peer-reviewed publications, ensuring that the synthesized evidence met established quality standards and methodological rigor, publication bias may have been introduced. This approach could underestimate null or negative findings and underrepresent evidence regarding the real-world feasibility and implementation barriers of CIMT, which are often reported in non-peer-reviewed sources such as theses or conference proceedings. Sixth, although overlap among meta-analyses was observed, pooled estimates were not combined, and results were interpreted separately to reduce the potential impact of duplicated primary studies. Seventh, although our eligibility criteria included any validated instrument based on ICF constructs, the findings are influenced by the predominance of MAL, FIM, and SS-QOL among the included systematic reviews. This may limit the representativeness of other validated but less frequently reported scales in the current synthesized literature on CIMT. These limitations highlight the need for multicenter studies with standardized designs and larger samples to confirm the results and establish more robust clinical recommendations. In addition to these limitations, certain strengths should be noted. Future standardized RCTs should prioritize (1) long-term follow-up (beyond 12 months) to assess the sustainability of gains; (2) the use of wearable sensors to objectively measure real-world limb use instead of relying solely on self-reports; and (3) cost-effectiveness analyses in public health systems.

This review evaluated multiple outcomes, including motor function, quality of movement, independence in ADLs and QoL, providing a comprehensive understanding of the impact of the intervention. In addition, this study examined the effectiveness of CIMT at different phases of stroke recovery, helping to identify the optimal timing for its application and thereby contributing valuable insights into clinical decision-making.

## 5. Conclusions

In summary, the findings of this umbrella review revealed the effectiveness of CIMT in improving motor function of the upper limb, as measured by the MAL scale, in adult patients after stroke. These improvements translate to enhanced performance ADLs and better QoL. CIMT is an intervention with considerable potential for effective combination with other therapies, such as motor imagery, virtual reality, and physiotherapy. However, further RCTs are needed in populations with diverse aetiologies and standardized intervention protocols, including consistent dosing and intensity, to facilitate the integration of this therapy into routine clinical practice.

## Figures and Tables

**Table 1:** Characteristics of the included systematic reviews.

N.I Study	Reference	Studies Included (n)	Type of Stroke and Phase of Development	Number of Subjects	Age Range (Years)	Intervention (EG)	Comparison (CG)	Duration (Weeks)	Study Variables	Assessment Tools	Quality Assessment—AMSTAR 2
1	Bonaiuti et al. (2007) [20]	9 RCTs	N. R	243 (EG: 113) (CG: 130)	N.R, 18–95 in some studies	CIMT	Conventional therapy	2–10	UL motor function, ADLs performance, treatment adherence	ARAT, MAL FMA, WMFT, BI, FIM	Low
2	Ekechukwu et al. (2020) [5]	347 RCTs	Ischemic and hemorrhagic, in acute, subacute, and chronic phases	N.R	N.R. adults + 18 years	CIMT, VR, videogames, NES, OT, robotic devices, task-based therapy	Conventional therapy	Variable	ADLs performance, gait, muscular strength, quality of life, motor function, spasticity, mobility	FMA, WMFT, ARAT, BBT, BI, MAL, FIM, SIS, 6-Minute Walk Test, 10-Meter Walk Test, SF-36	Moderate
3	French et al. (2016) [2]	33 RCTs	Ischemic and hemorrhagic, in acute, subacute and chronic phases	1853 (EG: 913) (CG: 940)	18-70	Task Repetitive Training	Usual care and/or no treatment	2-20	UL and LL motor function, ADLs performance, quality of life, global motor function	ARAT, WMFT, MAS, MAL, 6 Minute Walk Test, Berg Balance Scale, Timed Up and Go Test, BI, FIM, SF-36, SIS	High
4	García-Rudolph et al. (2019) [21]	31 RCTs	Ischemic and hemorrhagic, in chronic phase	25275 (EG: 12589) (CG: 12686)	N.R, adults + 18 years	CIMT, NES, robotic devices, task-based therapy	Standard care, usual care, or no treatment	Variable	ADLs performance, mobility, UL motor function, quality of life and functional recovery	BI, FIM, MAL, SS-QOL	Moderate
5	Hakkenees y Keating (2005) [22]	14 RCTs	Ischemic and hemorrhagic, in acute, subacute and chronic phases	N.R	N.R, adults + 18 years	CIMT, mCIMT	Standard care, alternative therapies, or no intervention	2–10	UL motor function, ADLs performance, quality of life	ARAT, WMFT, FMA, MAL, FIM	Moderate
6	Hatem et al. (2016) [8]	270 RCTs	Ischemic and hemorrhagic, in acute, subacute and chronic phases	N.R	N.R, + 18 years	CIMT, mirror therapy, robotics, VR, and brain stimulation	Conventional therapy	2–26	ADLs performance, functional disability, motor function UE	FMA, MAL, ARAT, SS-QOL	Moderate
7	Hussain et al. (2022) [23]	16 RCTs	Ischemic and hemorrhagic in acute, subacute and chronic phases	865 (EG: 499) (CG: 366)	30–85	Task-oriented rehabilitation	Conventional or noninterventional therapy	2–6	UL motor function, ADLs performance, quality of life	FMA, WMFT, BBT, MAS	Moderate
8	Pollock et al. (2014) [1]	40 RCTs	Ischemic and hemorrhagic, in acute, subacute and chronic phases	18078 (EG: 9017) (CG: 9061)	N.R, + 18 years	CIMT, mental practice, mirror therapy, VR, electrical stimulation, unilateral and bilateral training	No intervention, conventional therapy, placebo or alternative intervention	Variable	UL functionality, ADLs performance, motor impairment and quality of life	ARAT, FMA, MAL, WMFT, BI	High
9	Pulman y Buckley (2013) [24]	22 RCTs	Ischemic and hemorrhagic, in subacute and chronic phases	1503 (EG: NR) (CG: NR)	23–83	CIMT, BTX, Task-oriented rehabilitation, robotic devices, mirror therapy, NES	Conventional or noninterventional therapy	2–52	Quality of life, motor function, strength, movility, ADLs performance, pain	SIS, Nottingham Health Profile, Sickness Impact Profile, SF-36, SS-QOL	Moderate

ARAT: Action Research Arm Test; ADLs: Activities Daily Living; BBT: Box and Block Test; BI: Barthel Index; BTX: Botulinum toxin; CG: Control Group; BI: Barthel Index; CIMT: Constraint Induced Movement Therapy; EG: Experimental Group; FIM: Lower Limb; MAL: Motor Activity Log; MAS: Modified Ashworth Scale; mCIMT: modified Constraint Induced Movement Therapy; NES: Neuromuscular Electrical Stimulation; N.R: Not reported; N.I: Number of identification; OT: Occupational Therapy; RCTs: Randomized Controlled Trials; SIS: Stroke Impact Scale; SF-36: Short Form Health Survey 36-item; SS-QOL: Stroke Specific Quality of Life Scale; UL: Upper Limb; VR: Virtual Reality; WMFT: Wolf Motor Function Test.

**Table 2:** Characteristics of the included meta-analyses.

N.I Study	Reference	Studies Included (n)	Type of Stroke and Phase of Development	Number of Subjects	Age Range (Years)	Intervention (GE)	Comparison (GC)	Duration (Weeks)	Study Variables	Assessment Tools	Quality Assessment—AMSTAR 2
10	Corbetta et al. (2015) [25]	42 RCTs	Ischemic and hemorrhagic, N.S phase	1453 (EG: 732) (CG: 721)	37–87	CIMT and mCIMT	Conventional physiotherapy treatment and/or no treatment	2–10	UL motor function, perception of use of affected arm, dexterity, quality of life, disability, ADL performance	BI, SIS, FMA, MAL	High
11	Corbetta et al. (2010) [10]	18 RCTs	Ischemic and hemorrhagic, N.S phase	674 (EG: N.S) (CG: N.S)	36–87	CIMT, mCIMT, FU	Conventional rehabilitation (Physiotherapy and/or OT) and/or absence of treatment	2–10	UL motor function, disability, ADLs performance	BI, FIM, ARAT, WMFT, MAS	High
12	De Azevedo et al. (2022) [26]	21 RCTs	Ischemic and hemorrhagic, in acute, subacute and chronic phases	N.R	18–90	CIMT, CIMT + Mirror therapy	Conventional therapy (Physiotherapy and OT)	2–10	UL motor function, ADLs performance, social participation	FMA, WMFT, ARAT, MBI, MAL, FIM	Moderate
13	Etoom et al. (2016) [27]	38 RCTs	Ischemic and hemorrhagic, in acute, subacute and chronic phases	1561 (EG: 769) (CG: 792)	18–87	CIMT and mCIMT	Conventional rehabilitation and/or no intervention	2–10	UL motor function, ADLs performance, functional mobility	FMA, MAL, WMFT, ARAT	Moderate
14	Gao et al. (2024) [7]	34 RCTs	Ischemic and hemorrhagic, in acute, subacute and chronic phases	2399 (EG: 1212) (CG: 1187)	18–87	CIMT, physical exercise, music therapy and art therapy	Standard care or no intervention	Variable	Quality of life; physical, mental, and social function; ADLs performance	SIS, SF-36, EQ-5D, SS-QOL	High
15	Hestetun-Mandrup et al. (2024) [28]	13 RCTs	Ischemic and hemorrhagic, in subacute and chronic phases	571 (EG: 290) (CG: 293)	N.R, +18 years	VR, CIMT, telerehabilitation apps	Conventional rehabilitation	2–8	Motor function, gait, quality of life	FMA, WMFT, MAS, BBS, MAL, SIS, SS-QOL	High
16	Jeon et al. (2015) [11]	11 RCTs	Ischemic and hemorrhagic, in acute, subacute and chronic phases	469 (EG: 239) (CG: 230)	N.R, +18 years	Task Repetitive Training	Standard care or no intervention	2–6	UL and LE motor function, walking speed and endurance, balance, muscle strength	TUG, BBS, FMA, 6-Minute Walk Test	Moderate
17	Kaneko et al. (2024) [6]	18 RCTs	Ischemic and hemorrhagic, in subacute and chronic phases	330 (EG: 167) (CG: 163)	54–60	CIMT + adjuvant therapies (mirror therapy, electrical stimulation, mental practice)	Standard CIMT	2–10	UL motor function, ADLs performance, quality of movement, perceived use of the affected arm	FMA, MAL, ARAT	High
18	Liu et al. (2017) [29]	16 RCTs	Ischemic and hemorrhagic, in acute and subacute phases	710 (EG: 370) (CG: 340)	N.R, +18 years	CIMT, mCIMT of high and low intensity	Traditional rehabilitation therapy	2–10	UL motor function, ADLs performance	FMA, ARAT, MAL, MBI, WMFT	Moderate
19	McIntyre et al. (2012) [4]	16 RCTs	Ischemic and hemorrhagic, in chronic phase	571 (EG: 266) (CG: 305)	30–87	CIMT, mCIMT	Traditional rehabilitation therapy or no intervention	2–10	UL motor function, motor recovery, ADLs performance	MAL, FMA, ARAT, WMFT, FIM	Moderate
20	Nijland et al. (2011) [9]	5 RCTs	Ischemic and hemorrhagic, in acute and subacute phases	106 (EG: 64) (CG: 42)	N.R, +18 years	CIMT, CIMT of low intensity	Conventional therapy or standard care	2–3	UL motor function, manual dexterity, perceived use of the affected arm, quality of movement	FMA, ARAT, MAL, GPT	Moderate
21	Peurala et al. (2012) [30]	27 RCTs	Ischemic and hemorrhagic, in acute, subacute and chronic phases	N.R	N.R, +18 years	CIMT, mCIMT	Conventional care or no intervention	2–10	UL motor function, self-care, ADLs performance	MAL, ARAT, WMFT, FIM, BI	Moderate
22	Pulman et al. (2013) [24]	6 RCTs	Ischemic and hemorrhagic, in subacute and chronic phases	612 (EG: 237) (CG: 375)	23–83	CIMT, conventional rehabilitation, Bilateral training	BTX-Type A	EG: 2–3 to 12 months CG: 12–24	Quality of life, strength, hand function, ADLs performance, participation, memory, communication	SIS, Stroke Adapted Sickness Impact Profile, European Quality of Life-5D	Moderate
23	Sanchez et al. (2024) [3]	2 RCTs	Ischemic and hemorrhagic, in chronic phase	109 (EG: 57) (CG: 52)	31–83	CIMT, telerehabilitation	Traditional CIMT	2–3	UL motor function, daily use of the affected arm	WMFT, MAL, Quality of Life in Neurological Conditions	High
24	Shi et al. (2011) [32]	13 RCTs	Ischemic and hemorrhagic, in acute, subacute and chronic phases	278 (EG: 143) (CG: 135)	31–83	mCIMT	Traditional rehabilitation (Physiotherapy and OT)	2–10	UL motor function, functional disability, perceived use of the affected arm, kinematic analysis of movement	FMA, ARAT, WMFT, FIM, BI, MAL, kinematic analysis	Moderate
25	Thrane et al. (2014) [33]	23 RCTs	Ischemic and hemorrhagic, in acute, subacute and chronic phases	1002 (EG: 519) (CG: 483)	48–71	CIMT, mCIMT	Traditional therapy	1–4	UL motor function, ADLs performance, participation	FMA, ARAT, MAL, WMFT, BI	Moderate

ARAT: Action Research Arm Test; ADLs: Activities Daily Living; BBT: Box and Block Test; BI: Barthel Index; BTX: Botulinum toxin; CG: Control Group; BI: Barthel Index; CIMT: Constraint-Induced Movement Therapy; EG: Experimental Group; FIM: Functional Independence Measure; MAL: Motor Activity Log; MAS: Modified Ashworth Scale; mCIMT: modified Constraint-Induced Movement Therapy; NES: Neuromuscular Electrical Stimulation; N.R: Not reported; N.I: Number of identification; OT: Occupational Therapy; RCTs: Randomized Controlled Trials; SIS: Stroke Impact Scale; SF-36: Short Form Health Survey 36-item; SS-QOL: Stroke Specific Quality of Life Scale; UL: Upper Limb; VR: Virtual Reality; WMFT: Wolf Motor Function Test.

## Data Availability

The datasets generated and analysed during the current study are available from the corresponding author upon reasonable request.

## Supplementary Materials

The following supporting information can be downloaded at: https://www.mdpi.com/article/10.3390/jcm15062451/s1, Table S1: PRISMA Checklist; Table S2: Search strategy; Table S3: Search equations in databases; Table S4: Assessment of the methodological quality of the included systematic reviews using the AMSTAR 2 tool; Table S5: Assessment of the methodological quality of the included meta-analyses using the AMSTAR 2 tool; Table S6: Quality grading of evidence for motor functionality in upper limbs; Table S7: Quality grading of evidence for activity of daily living performance.; Table S8: Quality grading of evidence for quality of life.

## Abbreviations

The following abbreviations are used in this manuscript:

ADLs	Activities of Daily Living
ARAT	Action Research Arm Test
BBT	Box and Block Test
BI	Barthel Index
BTX	Botulinum Toxin
CCA	Corrected Covered Area
CGs	Control Groups
CIMT	Constraint-Induced Movement Therapy
CIs	Confidence Intervals
EGs	Experimental Groups
FIM	Functional Independence Measure
FMA	Fugl Meyer Assessment
GRADE	Grading of Recommendations, Assessment, Development, and Evaluation
LL	Lower Limb
MAL	Motor Activity Log
MAS	Modified Ashworth Scale
mCIMT	modified Constraint-Induced Movement Therapy
NES	Neuromuscular Electrical Stimulation
NI	Number of Identification
NR	Non-Reported
OT	Occupational Therapy
PRISMA	Preferred Reporting Items for Systematic Reviews and Meta-Analysis
QoL	Quality of Life
RCTs	Randomized Controlled Trials
SF-12	Short Form-12
SF-36	Short Form-36
SIS	Stroke Impact Scale
SMDs	Standard Measure Differences
SS-QOL	Stroke Specific Quality of Life Scale
UL	Upper Limb
VR	Virtual Reality
WMFT	Wolf Motor Function Test
