# Supplementary material for: Constraint-Induced Movement Therapy in the Rehabilitation of Adults After Stroke: An Umbrella Review
Source: J Clin Med. 2026 Mar 23;15(6):2451. doi: 10.3390/jcm15062451 (PMC13028200; doi:10.3390/jcm15062451)
Supplement: Supplementary file 1 [file jcm-15-02451-s001.zip › jcm-4187910-supplementary.pdf]

SUPPLEMENTARY MATERIAL

Table S2. Search strategy.

Component		Description
**Population**	**P**	Stroke / chronic stroke / subacute stroke, hemiparesis, upper limb, upper extremity
**Intervention**	**I**	Constraint induced movement therapy, repetitive task practice
**Comparator**	**C**	Not specified
**Outcomes**	**O**	Quality of life, motor activity, participation, activity daily living, motor ability, motor function

Table S3. Search equations in databases.

Database	Keywords	Search equation	Articles	Date
**Pubmed**	Stroke, "chronic stroke", "subacute stroke", hemiparesis, upper limb, upper extremity, "Constraint induced movement	(("Stroke"[Mesh] OR "Stroke"[Title/Abstract] OR "Hemiparesis"[Title/Abstract] OR "Paresis"[Mesh]) AND ("Upper Extremity"[Mesh] OR "Upper Limb"[Title/Abstract])) AND ("Constraint-Induced Therapy"[Mesh] OR "Constraint-Induced Movement Therapy"[Title/Abstract] OR "CIMT"[Title/Abstract] OR "Repetitive Task Practice"[Title/Abstract]) AND ("Quality of Life"[Mesh] OR "Activities of Daily Living"[Mesh] OR "ADL"[Title/Abstract] OR "Patient Participation"[Mesh] OR "Motor Skills"[Mesh]) AND (systematicreview[Filter] OR metaanalysis[Filter])	19	15-03-2026

	therapy", "repetitive task practice", CIMT, Quality of life, motor activity, participation, activity daily living, motor function			
**Web of Science**	Stroke, "chronic stroke", "subacute stroke", hemiparesis, upper limb, upper extremity, "Constraint induced movement therapy", "repetitive task practice", CIMT, Quality of life, motor activity, participation, activity daily living, motor function	("Stroke" OR "Hemiparesis" OR "Paresis") AND ("Upper Extremity" OR "Upper Limb") AND ("Constraint-Induced Therapy" OR "Constraint-Induced Movement Therapy" OR "CIMT" OR "Repetitive Task Practice") AND ("Quality of Life" OR "Activities of Daily Living" OR "ADL" OR "Patient Participation" OR "Motor Skills") AND (Systematic review OR metanalysis)	50	15-03-2026
**Scopus**	Stroke, "chronic stroke", "subacute stroke", hemiparesis, upper limb, upper extremity, "Constraint induced movement therapy", "repetitive task practice", CIMT,	TITLE-ABS-KEY (("Stroke" OR "Hemiparesis" OR "Paresis") AND ("Upper Extremity" OR "Upper Limb") AND ("Constraint-Induced Therapy" OR "Constraint-Induced Movement Therapy" OR "comt" OR "Repetitive Task Practice") AND ("Quality of Life" OR "Activities of Daily Living" OR "ADL" OR "Patient Participation" OR "Motor Skills") AND (Systematic review OR methanalysis))	40	15-03-2026

	Quality of life, motor activity, participation, activity daily living, motor function	

Table S4. Assessment of the methodological quality of the included systematic reviews using the AMSTAR 2 tool.

**Reference**	**1**	**2**	**3**	**4**	**5**	**6**	**7**	**8**	**9**	**10**	**11**	**12**	**13**	**14**	**15**	**16**	**Risk of bias**
Bonaiuti et al. (2007)	Y	N	Y	PY	Y	Y	Y	Y	Y	N	N	N	Y	N	Y	N	Low
Ekechukwu et al. (2020)	PY	N	Y	Y	PN	PN	N	Y	PY	N	N	N	PY	PY	N	Y	Moderate
French et al. (2016)	Y	Y	Y	Y	Y	Y	Y	Y	Y	N	Y	Y	Y	Y	Y	N	High
García-Rudolph et al. (2019)	Y	N	Y	PY	Y	Y	Y	Y	Y	N	N	N	Y	N	Y	N	Moderate
Hakkenees y Keating (2005)	Y	N	Y	PY	Y	Y	Y	Y	Y	N	N	N	Y	N	Y	N	Moderate
Hatem et al. (2016)	Y	N	Y	PY	Y	Y	Y	Y	Y	N	N	N	Y	Y	Y	N	Moderate
Hussain et al. (2022)	Y	N	Y	Y	Y	Y	Y	Y	Y	N	Y	N	Y	N	Y	N	Moderate
Pollock et al. (2014)	Y	Y	Y	Y	Y	Y	Y	Y	Y	Y	Y	Y	Y	Y	Y	N	High
Pulman y Buckley (2013)	Y	N	Y	Y	PN	PY	N	Y	Y	N	N	N	PY	PY	N	Y	Moderate

Y: yes; N: no; PN: partial no; PY: partial yes. 1) Did the research questions and inclusion criteria for the review include the components of PICO? 2) Did the report of the review contain an explicit statement that the review methods were established prior to the conduct of the review and did the report justify any significant deviations from the protocol? 3) Did the review authors explain their selection of the study designs for inclusion in the review? 4) Did the review authors use a comprehensive literature search strategy? 5) Did the review authors perform study selection in duplicate? 6) Did the review authors perform data extraction in duplicate? 7) Did the review authors provide a list of excluded studies and justify the exclusions? 8) Did the review authors describe the included studies in adequate detail? 9) Did the review authors use a satisfactory technique for assessing the risk of bias (RoB) in individual studies that were included in the review? 10) Did the review authors report on the sources of funding for the studies included in the review? 11) If meta-analysis was performed did the review authors use appropriate methods for statistical combination of results? 12) If meta-analysis was performed, did the review authors assess the potential impact of RoB in individual studies on the results of the meta-analysis or other evidence synthesis? 13) Did the review authors account for RoB in individual studies when interpreting/ discussing the results of the review? 14) Did the review authors provide a satisfactory explanation for, and discussion of, any heterogeneity observed in the results of the review? 15) If they performed quantitative synthesis did the review authors carry out an adequate investigation of publication bias (small study bias) and discuss its likely impact on the results of the review? 16) Did the review authors report any potential sources of conflict of interest, including any funding they received for conducting the review?

Table S5. Assessment of the methodological quality of the included meta-analyses using the AMSTAR 2 tool.

**Referencia**	**1**	**2**	**3**	**4**	**5**	**6**	**7**	**8**	**9**	**10**	**11**	**12**	**13**	**14**	**15**	**16**	**Risk of bias**
Corbetta et al. (2015)	Y	N	Y	PY	Y	Y	Y	Y	Y	N	Y	Y	Y	Y	Y	N	High
Corbetta et al. (2010)	Y	N	Y	Y	Y	Y	Y	Y	Y	N	Y	N	Y	Y	Y	Y	High
De Azevedo et al. (2022)	Y	N	Y	Y	Y	Y	Y	Y	Y	N	Y	N	Y	PN	Y	N	Moderate
Etoom et al. (2016)	Y	N	Y	PY	Y	Y	Y	Y	Y	N	N	N	Y	Y	Y	N	Moderate
Gao et al. (2024)	Y	Y	Y	Y	Y	Y	Y	Y	Y	N	Y	N	Y	Y	Y	N	High
Hestetun-Mandrup et al. (2024)	Y	Y	Y	Y	Y	Y	Y	Y	Y	PY	Y	Y	Y	Y	Y	Y	High
Jeon et al. (2015)	Y	N	Y	PY	Y	Y	Y	Y	Y	N	Y	N	Y	Y	Y	N	Moderate

Kaneko et al. (2024)	Y	Y	Y	Y	Y	Y	Y	Y	Y	N	Y	N	Y	Y	Y	N	High
Liu et al. (2017)	Y	N	Y	Y	Y	Y	Y	Y	Y	N	Y	N	Y	Y	Y	N	Moderate
McIntyre et al. (2012)	Y	N	Y	PY	Y	Y	Y	Y	Y	N	Y	N	Y	N	Y	N	Moderate
Nijland et al. (2011)	Y	N	Y	PY	Y	Y	Y	Y	Y	N	Y	N	Y	Y	Y	N	Moderate
Peurale et al. (2012)	Y	N	Y	PY	Y	Y	Y	Y	Y	N	Y	N	Y	Y	Y	N	Moderate
Pulman et al. (2013)	Y	N	Y	Y	PN	PY	N	Y	Y	N	Y	PY	Y	Y	N	Y	Moderate
Sanchez et al. (2024)	Y	Y	Y	Y	Y	Y	Y	Y	Y	Y	Y	Y	Y	Y	Y	N	High
Shi et al. (2011)	Y	N	Y	Y	Y	Y	Y	Y	Y	N	Y	N	Y	Y	Y	N	Moderate
Thrane et al. (2014)	Y	N	Y	Y	Y	Y	Y	Y	Y	N	Y	N	Y	Y	Y	N	Moderate

Y: yes; N: no; PN: partial no; PY: partial yes. 1) Did the research questions and inclusion criteria for the review include the components of PICO? 2) Did the report of the review contain an explicit statement that the review methods were established prior to the conduct of the review and did the report justify any significant deviations from the protocol? 3) Did the review authors explain their selection of the study designs for inclusion in the review? 4) Did the review authors use a comprehensive literature search strategy? 5) Did the review authors perform study selection in duplicate? 6) Did the review authors perform data extraction in duplicate? 7) Did the review authors provide a list of excluded studies and justify the exclusions? 8) Did the review authors describe the included studies in adequate detail? 9) Did the review authors use a satisfactory technique for assessing the risk of bias (RoB) in individual studies that were included in the review? 10) Did the review authors report on the sources of funding for the studies included in the review? 11) If meta-analysis was performed did the review authors use appropriate methods for statistical combination of results? 12) If meta-analysis was performed, did the review authors assess the potential impact of RoB in individual studies on the results of the meta-analysis or other evidence synthesis? 13) Did the review authors account for RoB in individual studies when interpreting/ discussing the results of the review? 14) Did the review authors provide a satisfactory explanation for, and discussion of, any heterogeneity observed in the results of the review? 15) If they performed quantitative synthesis did the review authors carry out an adequate investigation of publication bias (small study bias) and discuss its likely impact on the results of the review? 16) Did the review authors report any potential sources of conflict of interest, including any funding they received for conducting the review?

Table S6. Quality grading of evidence for Motor Functionality in upper limbs.

Certainty assessment							№ of patients		Effect		Certainty	Importance
**№ of participants**	**Study design**	**Risk of bias**	**Inconsistency**	**Indirectness**	**Imprecision**	**Other considerations**	**GE**	**GC**	**Relative (95% CI)**	**Absolute (95% CI)**		
**Corbetta et al. (2015)**												
42	randomised trial	serious [a,b]	not serious [k]	Serious [g]	Serious [e,f]	Publication bias is strongly suspected [a]	732	721	-	SMD **0.79 SD higher.** (0.5 higher. to 1.08 higher.)	⊕◯◯◯ Very low [a,b,e,f,g,k]	CRITICAL
**Corbetta et al. (2010)**												
18	randomised trial	serious [b,e]	Serious [g]	Serious [g]	Serious [e,f]	Publication bias is strongly suspected [e]			-	SMD **0.44 SD higher.** (0.03 higher. to 0.84 higher.)	⊕◯◯◯ Very low [b,e,f,g]	IMPORTANT
**De Azevedo et al. (2022)**												
21	randomised trial	serious [a,b]	very serious [i]	Serious [g]	Serious [e,f]	Publication bias is strongly suspected [e]			-	SMD **0.53 SD higher.** (0.4 higher. to 0.66 higher.)	⊕◯◯◯ Very low [a,b,e,f,g,i]	CRITICAL
**Etoom et al. (2016)**												

38	randomised trial	Very serious [b,e]	not serious [k]	Serious [g]	Serious [e,f]	Publication bias is strongly suspected [e]	769	792	-	SMD **0.56 SD higher.** (0.3 higher. to 0.81 higher.)	⊕◯◯◯ Very low [b,e,f,g,k]	CRITICAL
**Hestetun-Mandrup et al. (2024)**												
13	randomised trial	serious [a,e]	very serious [g]	Serious [d]	Serious [e,f]	Publication bias is strongly suspected [e]	290	293	-	SMD **0,09 SD higher.** (0.15 minor to 0,34 higher.)	⊕◯◯◯ Very low [a,d,e,f,g]	CRITICAL
**Jeon et al. (2015)**												
11	randomised trial	serious [a,b,e]	very serious [i]	Serious [g]	Serious [e,f]	Publication bias is strongly suspected [b,e,g]	239	230	-	SMD **1.52 SD higher.** (1.08 minor to 3.82 higher.)	⊕◯◯◯ Very low [a,b,e,f,g,i]	CRITICAL
**Kaneko et al. (2024)**												
18	randomised trial	serious [a,e]	very serious [i]	Serious [g]	Serious [e,f]	Publication bias is strongly suspected [a,e]	167	163	-	SMD **4.02 SD higher.** (2.6 higher.	⊕◯◯◯ Very low [a,e,f,g,i]	CRITICAL

										to 5.44 higher.)		
**Liu et al. (2017)**												
16	randomised trial	very serious [a,b]	Serious [c]	very serious [a,l]	Serious [e,f]	Publication bias is strongly suspected [b]	370	340	-	SMD **0.81 SD higher.** (0.33 higher. to 1.29 higher.)	⊕◯◯◯ Very low [a,b,c,e,f,l]	CRITICAL
**McIntyre et al. (2012)**												
16	randomised trial	serious [b]	not serious [k]	Serious [d]	Serious [e,f]	Publication bias is strongly suspected [a,e]	266	305	-	SMD **0.13 SD higher.** (0.02 higher. to 0.37 higher.)	⊕◯◯◯ Very low [a,b,d,e,f,k]	IMPORTANT
**Nijland et al. (2011)**												
5	randomised trial	serious [b,e,g]	very serious [i]	Serious [a,g]	Serious [e,f]	Publication bias is strongly suspected [a,b,e,g]	64	42	-	SMD **1.15 SD higher.** (0.33 minor to 2.62 higher.)	⊕◯◯◯ Very low [a,b,e,f,g,i]	CRITICAL
**Peurale et al. (2012)**												
27	randomised trial	serious [a,e]	Serious [c]	Serious [d]	Serious [e,f]	Publication bias is strongly suspected [a,d,e]			-	SMD **0.85 SD higher.**	⊕◯◯◯ Very low [a,c,d,e,f]	CRITICAL

										(0.62 higher. a 1.08 higher.)		
**Sanchez et al. (2024)**												
2	randomised trial	not serious [h]	not serious [k]	Serious [d]	Serious [e,f]	none	57	52	-	SMD **0.33 SD higher.** (0.04 minor to 0.42 higher.)	⊕⊕◯◯ Low [d,e,f,h,k]	IMPORTANT
**Shi et al. (2011)**												
13	randomised trial	Not serious [h]	Serious [c]	very serious [l]	Serious [e,f]	none	143	135	-	SMD **1.41 SD higher.** (1.07 higher. a 1.76 higher.)	⊕◯◯◯ Very low [c,e,f,h,l]	CRITICAL
**Thrane et al. (2014)**												
23	randomised trial	serious [b,d]	Serious [c]	Serious [a,d]	Serious [e,f]	Publication bias is strongly suspected [a,b,d,e]	519	483	-	SMD **0.51 SD higher.** (0.3 higher. a 0.73 higher.)	⊕◯◯◯ Very low [a,b,c,d,e,f]	CRITICAL

CI: Confidence interval; **SMD:** Standard mean difference

Explanations

a. Some concerns

b. High risk of bias

c. I^2= 50 - 75 %

d. Moderate heterogeneity

e. Small number of studies

f. Wide confidence intervals

g. Considerable heterogeneity

h. Non reported

i. I^2 = + 75 %

j. Egger's test statistically significant

k. I^2= 0 - 50 %

l. Substantial heterogeneity

Table S7. Quality grading of evidence for Activity of Daily Living Performance.

Certainty assessment							№ of patients		Effect			
**№ of participants**	**Study design**	**Riask of bias**	**Inconsistency**	**Indirectness**	**Imprecision**	**Other considerations**	**GE**	**GC**	**Relative (95% CI)**	**Absolute (95% CI)**	**Certainty**	**Importance**
**De Azevedo et al. (2022)**												
21	randomised trial	serious [a,e,k]	Serious [h]	Serious [l]	Serious [d,e]	Publication bias is strongly suspected [a,e,k,l]			-	SMD **5.44 SD higher.** (2.31 higher. to	⊕◯◯◯ Very low [a,d,e,h,k,l]	CRITICAL

										8.57 higher.)		
**Gao et al. (2024)**												
34	randomised trial	serious [c]	Serious [h]	Serious [c,f]	Serious [d,e]	Publication bias is strongly suspected [c]	1212	1187	-	SMD **10.42 SD higher.** (2.98 higher. to 17.87 higher.)	⊕◯◯◯ Very low [c,d,e,f,h]	CRITICAL
**Kaneko et al. (2024)**												
18	randomised trial	serious [a,k]	very serious [b]	Serious [c]	Serious [d,e]	Publication bias is strongly suspected [a,c,e]	167	163	-	SMD **0.15 SD higher.** (0.01 minor to 0.32 higher.)	⊕◯◯◯ Very low [a,b,c,d,e,k]	IMPORTANT
**Liu et al. (2017)**												
16	randomised trial	serious [a,k,l]	very serious [b]	Serious [l]	Serious [d,e]	Publication bias is strongly suspected [a,k,l]	370	340	-	SMD **10.71 SD higher.** (4.42 higher. to 16.97 higher.)	⊕◯◯◯ Very low [a,b,d,e,k,l]	CRITICAL
**McIntyre et al. (2012)**												
16	randomised trial	serious [a,k]	not serious [m]	Serious [c]	Serious [d,e]	Publication bias is strongly suspected [a,c,e,k]	266	305	-	SMD **1.7 SD higher.** (0.25 minor to 6.45 higher.)	⊕◯◯◯ Very low [a,c,d,e,k,m]	CRITICAL
**Pulman et al. (2013)**												
6	randomised trial	serious [a,c]	Serious [h]	Serious [c]	Serious [d,e]	Publication bias is strongly suspected [a,c]	237	375	-	SMD **0.03 SD higher.**	⊕◯◯◯ Very low [a,c,d,e,h]	IMPORTANT

										(0.094 minor to 0.164 higher.)		
**Shi et al. (2011)**												
13	randomised trial	serious [a,f]	Serious [h]	very serious [i]	Serious [d,e]	Publication bias is strongly suspected [a,c,k]	143	135	-	SMD **7 SD higher.** (5.75 higher. to 13.26 higher.)	⊕◯◯◯ Very low [a,c,d,e,f,h,i,k]	CRITICAL
**Thrane et al. (2014)**												
23	randomised trial	serious [a,c,k]	Serious [h]	Serious [c]	Serious [d,e]	Publication bias is strongly suspected [a,c,k]	519	483	-	SMD **0.23 SD higher.** (0.24 minor to 0.71 higher.)	⊕◯◯◯ Very low [a,c,d,e,h,k]	IMPORTANT

CI: Confidence interval; **SMD:** Standard mean difference

Explanations

a. Some concerns

b. I^2= + 75 %

c. Moderate heterogeneity

d. Wide confidence intervals

e. Small number of studies

f. Non reported

g. Low risk of bias

h. I^2 = 50 - 75 %

i. Substantial heterogeneity

j. Egger´s test statistically significant

k. High risk of bias

l. Considerable heterogeneity

m. I^2= 0 - 50 %

Table S8. Quality grading of evidence for Quality of Life.

Certainty assessment							№ of patients		Effect		Certainty	Importance
**№ of participants**	**Study design**	**Risk of bias**	**Inconsistency**	**Indirectness**	**Imprecision**	**Other considerations**	**GE**	**GC**	**Relative (95% CI)**	**Absolute (95% CI)**		
**Corbetta et al. (2015)**												
42	randomised trial	serious [a]	Serious [f]	Serious [g]	Serious [d,e]	Publication bias is strongly suspected [a,g]	732	721	-	SMD **6.54 SD higher.** (1.2 minor to 14.28 higher.)	⊕◯◯◯ Very low [a,d,e,f,g]	CRITICAL
**Gao et al. (2024)**												
34	randomised trial	serious [g]	Serious [f]	not serious [h]	Serious [d,e]	Publication bias is strongly suspected [g,h]	1212	1187	-	SMD **0.75 SD higher.** (0.31 higher. to 1.2 higher.)	⊕◯◯◯ Very low [d,e,f,g,h]	CRITICAL
**Hestetun-Mandrup et al. (2024)**												
13	randomised trial	serious [a]	Serious [f]	Serious [g]	Serious [d,e]	Publication bias is strongly suspected [a,d,g]	290	293	-	SMD **0.15 SD higher.**	⊕◯◯◯ Very low [a,d,e,f,g]	IMPORTANT

										(0.23 minor. to 0.53 higher.)		
**Pulman et al. (2013)**												
6	Randomised trial	serious [a]	Serious [f]	Serious [h]	Serious [d,e]	Publication bias is strongly suspected [a,d]	237	375	-	SMD **0.04 SD higher.** (0.065 minor to 0.138 higher.)	⊕◯◯◯ Very low [a,d,e,f,h]	IMPORTANT

CI: Confidence interval; **SMD:** Standard mean difference

Explanations

a. Some concerns

b. High risk of bias

c. Considerable heterogeneity

d. Small number of studies

e. Wide confidence intervals

f. I^2 = 50 - 75 %

g. Moderate heterogeneity

h. Non reported

i. Egger's test statistically significant

j. I^2 = + 75 %
